# Effect of TiO_2_ on Pd/La_2_O_3_-CeO_2_-Al_2_O_3_ Systems during Catalytic Oxidation of Methane in the Presence of H_2_O and SO_2_

**DOI:** 10.3390/ma16206784

**Published:** 2023-10-20

**Authors:** Ralitsa Velinova, Silviya Todorova, Daniela Kovacheva, Hristo Kolev, Yordanka Karakirova, Pavel Markov, Katerina Tumbalova, Georgi Ivanov, Anton Naydenov

**Affiliations:** 1 Institute of General and Inorganic Chemistry, Bulgarian Academy of Sciences, Acad. G. Bonchev Str., bl. 11, 1113 Sofia, Bulgaria; raligeorgieva@svr.igic.bas.bg (R.V.); didika@svr.igic.bas.bg (D.K.); pvlmarkov@svr.igic.bas.bg (P.M.); katerinatumbalova@mail.bg (K.T.); geoivanov@yahoo.com (G.I.); 2 Institute of Catalysis, Bulgarian Academy of Sciences, Acad. G. Bonchev Str., bl. 11, 1113 Sofia, Bulgaria; todorova@ic.bas.bg (S.T.); hgkolev@ic.bas.bg (H.K.); daniepr@ic.bas.bg (Y.K.)

**Keywords:** methane oxidation, Pd/La_2_O_3_-CeO_2_-TiO_2_-Al_2_O_3_, deactivation, sulfur poisoning, catalyst regeneration, TiO_2_

## Abstract

New results on the effect of TiO_2_ on Pd/La_2_O_3_-CeO_2_-Al_2_O_3_ systems for catalytic oxidation of methane in the presence of H_2_O and SO_2_ have been received. Low-temperature N_2_-adsorption, XRD, SEM, HRTEM, XPS, EPR and FTIR techniques were used to characterize the catalyst. The presence of Ce^3+^ on the catalytic surface and in the volume near the lantana was revealed by EPR and XPS. After aging, the following changes are observed: (i) agglomeration of the Pd-clusters (from 8 nm to 12 nm); (ii) transformation of part of the TiO_2_ from anatase to larger particles of rutile; and (iii)—the increase in PdO/Pd—ratio above its optimum. The modification by Ti of the La_2_O_3_-CeO_2_-Al_2_O_3_ system leads to higher resistance towards the presence of SO_2_ most likely due to the prevailing formation of unstable surface sulfites instead of thermally stable sulfates. Based on kinetic model calculations, the reaction pathway over the Pd/La_2_O_3_-CeO_2_-TiO_2_-Al_2_O_3_ catalyst follows the Mars–van Krevelen mechanism. For evaluation of the possible practical application of the obtained material, a sample of Pd/La_2_O_3_-CeO_2_-TiO_2_-Al_2_O_3_, supported on rolled aluminum-containing stainless steel (Aluchrom VDM^®^), was prepared and tested. Methane oxidation in an industrial-scale monolithic reactor was simulated using a two-dimensional heterogeneous reactor model.

## 1. Introduction

Methane is the main component of natural gas and recent investigations indicate that it has an even more significant impact on global warming than previously thought [1,2]. The sources of pollution by methane are agriculture landfills and the combustion of coal and natural gas [3]. Recently, the need for methane incineration is also due to problems arising from the production of electricity and the great concern regarding the protection of the environment [4,5,6,7,8].

A very effective technology for reducing methane emissions is catalytic combustion; however, its major drawback is connected with the deactivation of the catalysts (mainly based on palladium). Among the catalysts used for complete oxidation, palladium supported on γ-Al_2_O_3_ stands out due to its large surface area and cost-effectiveness [9,10,11]. It is known that PdO/Al_2_O_3_ catalysts are unstable at high temperatures, resulting in reduced alumina surface area and the transformation of palladium oxide into Pd^0^ [12,13]. Deactivation is also accelerated by palladium sintering at elevated temperatures and the presence in the gases of sulfur compounds and water vapor at high concentrations. Therefore, there is a pressing need to develop catalysts that not only exhibit high activity but also demonstrate excellent thermal stability and resistance to water and sulfur compounds. To enhance the properties of palladium-based catalysts, various additives have been explored.

Lanthanum, for instance, has been commonly used to prevent the deactivation of catalysts by retarding the conversion of palladium oxide into palladium and improving stability against alumina surface area loss [5,9].

As reported by Ozawa et al. [14], adding La_2_O_3_ stabilizes the surface area of alumina and further modification by CeO_2_ prevents the transformation of palladium oxide to palladium. It was suggested that the addition of La into CeO_2_ decreases the particle size and, as a result, inhibits the sintering of CeO_2_. In general, Ce–La-based compounds exhibit excellent catalytic performance due to the remarkable oxygen storage capacity; however at elevated temperatures (above 1000 °C), CeO_2_ sinters result in catalyst deactivation [11]. In our previous study [15], we demonstrated the high activity and thermal stability of the Pd/ La_2_O_3_-CeO_2_-Al_2_O_3_ catalyst; however, its resistance to sulfur dioxide can be considered unsatisfactory.

The modification by TiO_2_ is based on the fact that TiO_2_ is only weakly and reversibly sulfated in the presence of SO_2_ and oxygen [16]. To enhance the sulfur resistance of catalysts, TiO_2_ as a material has been introduced into exhaust gas catalysts [17,18]. The use of TiO_2_ aims to promote sulfur desorption and significant progress in sulfur tolerance is observed [18]. Its employment as a catalytic support is associated with increased activity due to phase–support interactions [19]. 

TiO_2_ can increase the mobility of oxygen by creating oxygen vacancies through a more efficient reduction process (from Ti^4+^ to Ti^3+^), supplying the Lewis acid sites for the adsorption and dissociation of molecules [20].

The three crystalline forms of TiO_2_ (anatase, rutile, and brookite) possess different properties, thus providing the possibility for the TiO_2_-supported catalysts to perform different catalytic behavior [21,22]. It should be pointed out that for environmental applications, anatase is the most frequently used [23]. The combination of TiO_2_, which acts as a scavenger for SO_2_ and H_2_O, along with inert SiO_2_, which facilitates the removal of poisoning compounds after exposure to SO_2_ and H_2_O, has proven effective in improving resistance to poisoning and the catalyst’s regenerative potential. This behavior reveals the Pd—TiO_2_-based catalyst could be an attractive material for further practical implementation [24]. 

Based on existing research, an effective catalyst for methane combustion should primarily consist of (i) γ—Al_2_O_3_ as a carrier matrix; (ii) Pd as a catalytically active component; (iii) La_2_O_3_ for thermal stabilization of γ—Al_2_O_3_; (iv) CeO_2_ to improve oxygen exchange to keep the optimal ratio between Pd and PdO; and (v) TiO_2_—for improving poisoning tolerance and catalyst regeneration. Regarding the choice of synthesis methods, it can be pointed out that the sol–gel method offers an advanced way to create solids with a high specific area, well-defined porosity, and high resistance to deactivation in a single step [25]. This technique permits the physical and chemical properties of the final solid to be controlled throughout the synthesis steps; thus, the method allows the incorporation of the catalytic active component during the gelation step and ensures effective metal–support interaction [26]. 

The current study aims to investigate a Pd/La_2_O_3_-CeO_2_-TiO_2_-Al_2_O_3_ model system prepared using the sol–gel method evaluation on catalytic activity, thermal stability, water vapor effect, sulfur resistance, and the possibility of use of the synthesized material as an active component for creating monolithic catalysts for application in reduction of methane emissions.

## 2. Materials and Methods

### 2.1. Catalysts Synthesis

The La_2_O_3_-CeO_2_-TiO_2_-Al_2_O_3_ support was synthesized via the sol–gel method by the procedure reported elsewhere [27]. The powdered Al[OCH(CH_3_)_2_]_3_ (≥98%, Sigma-Aldrich, St. Louis, MO, USA) was dissolved in water, and after that, amounts of 1M HNO_3_, cerium (III) nitrate hexahydrate (99%, Sigma-Aldrich, St. Louis, MO, USA), lanthanum(III) nitrate hexahydrate (≥99.9%, Sigma-Aldrich) and titanium(IV) isopropoxide (97%, Sigma-Aldrich, St. Louis, MO, USA) were included to produce a mixture containing 80 wt.% aluminum oxide, 8 wt.% cerium (IV) oxide, 4 wt.% lanthnum(III) oxide and 8% titanium dioxide. The prepared mixture was homogenized at 373 K, then refluxed for 72 h and the obtained gel was dried at ambient temperature before heat treatment in air for 4 h at 500 °C. The synthesized catalytic support was impregnated with an aqueous solution of palladium(II) nitrate hydrate (99.8%, Thermo Scientific Chemicals, Waltham, MA, USA) and treated for 2 h in 0.1 vol.% CH_4_ in N_2_ gas mixture at 450 °C (in the absence of O_2_) to produce a catalyst with a nominal palladium content of 2.0% (*w*/*w*).

In order to obtain data approaching the operation of the catalyst in the form of a monolithic catalytic element, experiments with Aluchrome VDM^®^ (VDM Metals International GmbH, Werdohl, Germany) were performed. The preparation of a single monolithic channel (D = 3.5 mm, L = 118 mm, thickness of 0.2 mm) involves the following stages: (i) thermal treatment of the alloy at 920 °C during 25 h, aiming at the formation of **α**-Al_2_O_3_ whiskers on the steel surface; (ii) application of a primary bonding layer of γ-Al_2_O_3_; (iii) coating with a sol containig La_2_O_3_-CeO_2_-TiO_2_-Al_2_O_3;_ and (iv) impregnation with palladium (II) nitrate dihydrate.

In the present study, the catalyst was denoted as Pd/La_2_O_3_-CeO_2_-TiO_2_-Al_2_O_3_.

### 2.2. Characterization Techniques

The physical nitrogen adsorption/desorption isotherms were examined at 77 K using a NOVA 1200e Surface & Pore Analyzer (Quantachrome, Boynton Beach, FL, USA). The Brunauer–Emmett–Teller (BET) equation was applied for a specific surface area estimation [28], the total pore volume being determined at a relative pressure of 0.99. The pore size distributions were determined using the desorption branch of the isotherms, employing the Barrett–Joyner–Halenda (BJH) method [29]. The volume of the micropores was derived by the V-t-method [30].

The X-ray diffraction (XRD) diffractograms of pure support as well as fresh, used, after sulfur poisoning and thermally aged catalysts were determined within the 2Θ range 10–80° on a Bruker D8 Advance diffractometer (Cu Kα radiation, LynxEye detector, Karlsruhe, Germany). The determination of phase composition was carried out using the EVA software package, which utilized the ICDD-PDF2(2014) database. To quantify and determine the average crystallite size, the Topas-4.2 program (Karlsruhe, Germany) was employed.

The surface morphology and elemental composition of the catalysts were examined on a scanning electron microscope SEM/FIB LYRA I XMU, TESCAN (Brno—Kohoutovice, Czech Republic) connected with an energy dispersive spectroscope (EDS) (QUANTAX 200, Bruker, Germany).

Transmission electron micrographs (TEM) were performed on a JEOL JEM 2100 mi-croscope (JEOL Ltd., Tokyo, Japan) operating at 200 kV. The catalyst specimens were sus-pended in ethanol via an ultrasonic bath and subsequently placed onto holey C/Cu grids.

The catalysts’ composition and electronic structure were examined using X-ray photoelectron spectroscopy (XPS). The chemical composition of the samples was measured by monitoring the areas and binding energies of C1s, O1s, La3d, Ce3d, Al2p, Pd3d, Ti2p, and S2p photoelectron peaks. The photoelectron peaks measurements have been carried out on the ESCALAB MkII (VG Scientific, now Thermo Scientific, Waltham, MA, USA) electron spectrometer. More about the setup of the spectrometer and data processing can be found in [31].

The Electron paramagnetic resonance (EPR) spectra were recorded by the JEOL JES-FA 100 EPR spectrometer. The spectrometer is equipped with a cylindrical resonator (TE011) and operates at X–band frequency (9.5 GHz). The catalysts were put in special quartz tubes and positioned in the cavity center. The EPR measurements were performed at temperatures from 123 to 323 K using a Varied Temperature Controller ES-DVT4 (JEOL Ltd., Tokyo, Japan). The low temperatures can be easily obtained by sending liquid nitrogen to the sample area. The following conditions were used: modulation frequency—100 kHz, microwave power 1 mW, modulation amplitude 0.2 mT, sweep 500 mT, time constant 0.3 s, and sweep time 2 min.

Fourier transform infrared spectroscopy (FTIR) was performed using a Nicolet 6700 FTIR spectrometer (Thermo Electron Corporation, Madison, WI, USA). The measurements were carried out in transmission mode, and the spectral resolution was set at 4 cm^−1^.

### 2.3. Catalytic Tests

The catalytic activity study was performed by using a flow reactor under the following specified conditions: catalyst volume of 0.7 cm^3^ (0.5 cm^3^ catalyst sample and 0.2 cm^3^ quartz–glass particles with the same size as the catalyst), irregular shaped particles with a diameter of 0.45 ± 0.15 mm. The inner reactor diameter is 6.0 mm. The gaseous hourly space velocity (GHSV_STP_) was 60,000 h^−1^. The inlet concentrations of reagents were varied as follows: CH_4_ concentrations: 5 × 10^−2^, 1.0 × 10^−1^ and 2.7 × 10^−1^ vol.%, O_2_ on levels of 0.9, 5.0 and 20.0 vol.%, additional H_2_O on levels of 0, 1.2 and 2.2 vol.%, and balance to 100 vol.% by N_2_ (4.6). The standard deviation of the experimental data was estimated based on six repeated measurements. Gas analysis was carried out by using of an on-line gas-analyzers for CO/CO_2_/O_2_ (Maihak-Sick Mod. S 710, V.1.31, Hamburg, Germany ), THC-FID (analyzer for total organic content in gas phase, Thermo FID-TG, SK Elektronik GmbH, Leverkusen, Germany) and for SO_2_ measurement (MultiGas FTIR Gas Analyzer 2030G, MKS Instruments Inc., Andover, MA, USA).

The catalytic activity study was performed by using a flow reactor under the following specified conditions: catalyst volume of 0.7 cm^3^ (0.5 cm^3^ catalyst sample and 0.2 cm^3^ quartz–glass particles with the same size as the catalyst), irregular shaped particles with a diameter of 0.45 ± 0.15 mm. The inner reactor diameter is 6.0 mm. The gaseous hourly space velocity (GHSV_STP_) was 60,000 h^−1^. The inlet concentrations of reagents were varied as follows: CH_4_ concentrations: 5 × 10^−2^, 1.0 × 10^−1^ and 2.7 × 10^−1^ vol.%, O_2_ on levels of 0.9, 5.0 and 20.0 vol.%, additional H_2_O on levels of 0, 1.2 and 2.2 vol.%, and balance to 100 vol.% by N_2_ (4.6). The standard deviation of the experimental data was estimated based on six repeated measurements. Gas analysis was carried out by using of an on-line gas-analyzers for CO/CO_2_/O_2_ (Maihak-Sick Mod. S 710, V.1.31, Hamburg, Germany ), THC-FID (analyzer for total organic content in gas phase, Thermo FID-TG, SK Elektronik GmbH, Leverkusen, Germany) and for SO_2_ measurement (MultiGas FTIR Gas Analyzer 2030G, MKS Instruments Inc., Andover, MA, USA).

## 3. Results and Discussion

### 3.1. Catalytic Experiments

**Figure 1:**
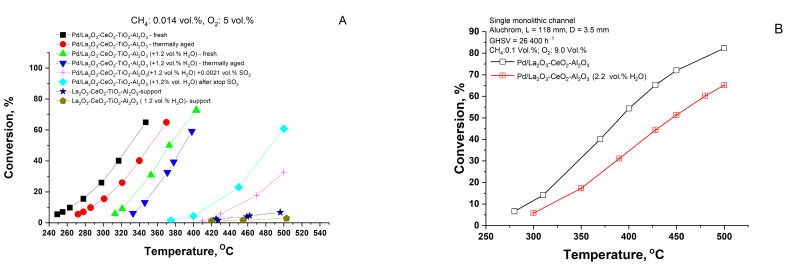
Catalytic activity of the Pd/La_2_O_3_-CeO_2_-TiO_2_-Al_2_O_3_ catalyst during the reaction of total methane oxidation in dry and humid gas feed, sulfur dioxide and after thermal aging (**A**) and tests on the Pd/La_2_O_3_-CeO_2_-TiO_2_-Al_2_O_3_ catalyst, prepared as single monolithic channel (**B**).

The data from the tests on total methane oxidation in the absence and presence of water vapor are shown in Figure 1. As can be seen, the light-off temperature (T_50_) in dry gas feed is about 328 °C, while in humid gas feed, the effect of the water leads to an increase of about 40 °C.

For possible practical application, two identical samples of the obtained catalyst were subjected to treatment in air as follows: (i) 170 h in the air at 500 °C in the absence and presence of 1.2 vol.% H_2_O (denoted thermally aged) and (ii) in the presence of sulfur dioxide (0.0021 vol.%) performed in the catalytic reactor for 48 h at 450 °C. It can be observed that after the thermal aging, the activity measured in dry gas feed shows a shift in T_50_ to higher temperatures of about 20 °C, while in the presence of 1.2 vol.% H_2_O it leads to further increase by another 40 °C. Data on the catalytic activity of La_2_O_3_-CeO_2_-TiO_2_-Al_2_O_3_ support in dry and humid gas feed are also represented for comparative analysis. It is evident that the pure support exhibits notably low catalytic activity. However, it is important to emphasize that its contribution should not be neglegted.

**Figure 2:**
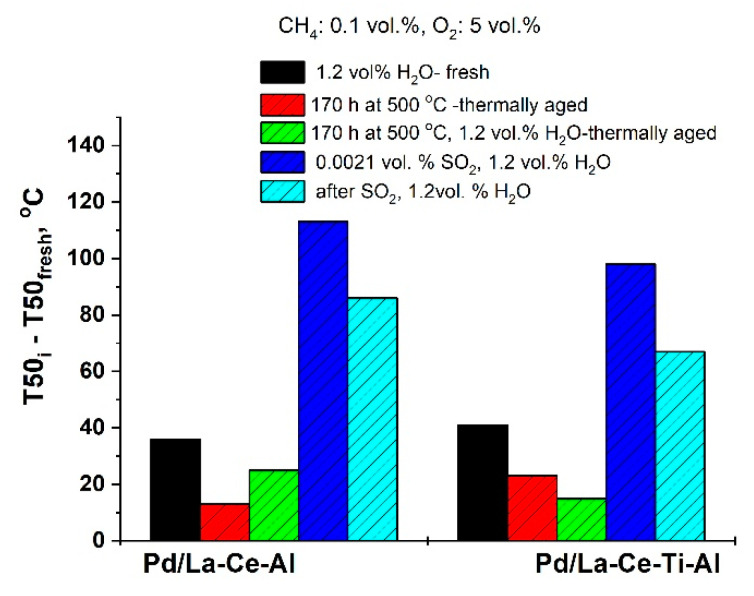
Comparative data on T_50_, measured with fresh samples and after tests for thermal aging and sulfur dioxide resistance.

The difference between the measured T_50_ for the fresh and for the corresponding stability tested Pd/La_2_O_3_-CeO_2_-Al_2_O_3_ and Pd/La_2_O_3_-CeO_2_-TiO_2_-Al_2_O_3_ samples are shown in Figure 2.

It is seen (Figure 2) that in humid gas feed, the effect of the water vapor in the Pd/La_2_O_3_-CeO_2_-TiO_2_-Al_2_O_3_ fresh sample is negligible when compared with the effect of the water on the Pd/La_2_O_3_-CeO_2_-Al_2_O_3_ sample. However, after the thermal aging, the activity of the Ti-containing sample in the humid gas has been improved, while in the dry gas its the activity is slightly lower as compared with the sample without Ti. Regarding sulfur deactivation, an increase in the temperatures (after the reaction in the presence of SO_2_) in T_50_ (apprx. 100–110 °C) was observed. Further testing without sulfur dioxide in the gas shows that the samples restore part of their initial activity, i.e., the Pd/La_2_O_3_-CeO_2_-TiO_2_-Al_2_O_3_ poses higher resistance to sulfur poisoning as compared with the Pd/La_2_O_3_-CeO_2_-Al_2_O_3_ sample.

Furthermore, the characterization of the catalyst was performed using a variety of methods.

### 3.2. Low-Temperature Nitrogen Adsorption

**Figure 3:**
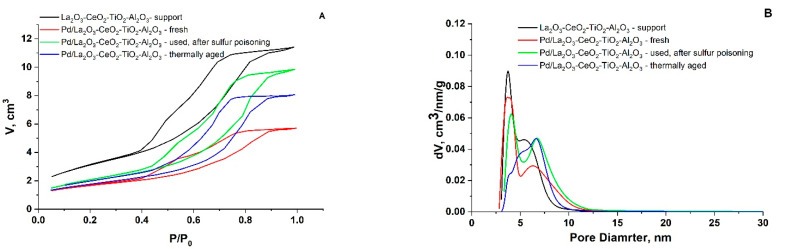
Adsorption/desorption isotherms (**A**) and PSD (**B**) of support and fresh, used, after sulfur poisoning and thermally aged catalysts.

The adsorption/desorption isotherm and pore size distribution (PSD) of the synthesized La_2_O_3_-CeO_2_-TiO_2_-Al_2_O_3_ support and the fresh, used, after sulfur poisoning and thermally aged catalysts prepared by the sol–gel method are presented in Figure 3A,B.

The obtained data indicate that all of the samples displayed similar characteristics, corresponding to a typical isotherm of type IV according to the IUPAC classification [32]. The prepared material is mesoporous of type H1 hysteresis, featuring clearly defined cylindrical-like pore channels or clusters of compact, nearly uniform spheres.

According to the BJH method from the desorption branch of the isotherms, the PSD was calculated (Figure 3B). All samples showed a bimodal structure. It was reported that support with a distinct bimodal pore structure shows significant benefits in catalytic reactions. This is attributed to the fact that the presence of large pores facilitates the molecular transport pathway, while the small pores offer a substantial surface area for supporting the active phase, as indicated in reference [33]. The samples exhibit a mesopore volume of approximately 0.2 cm^3^/g, with an average pore diameter ranging from 4.9 nm to 8.1 nm, depending on the applied test conditions (Table 1). The specific surface area is significantly decreased and the average pore diameter is increased as a result of the Pd deposition.

For evaluation of the possible extent of micropores, the V-t method has been applied. The external surface area, denoted as a S_ext_ of the microporous samples, was determined through the slope of the t-plot. Consequently, the micropore surface area (S_micro_) was calculated using the formula S_micro_ = S_BET_ − S_ext_. The obtained results show no significant presence of micropores for the used, after sulfur poisoning and thermally aged catalysts. Therefore, the reported data are based on the assumption that the total (BET) surface area is practically equal to the external surface (V-t method).

### 3.3. Powder X-ray Diffraction

**Figure 4:**
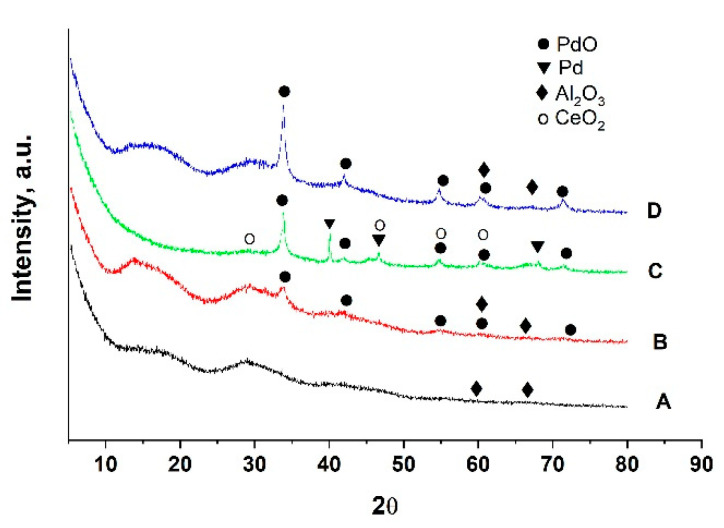
XRD patterns of La_2_O_3_-CeO_2_-TiO_2_-Al_2_O_3_ support (A) Pd/La_2_O_3_-CeO_2_-TiO_2_-Al_2_O_3_-fresh (B); Pd/La_2_O_3_-CeO_2_-TiO_2_-Al_2_O_3_-used, after sulfur poisoning (C) and Pd/La_2_O_3_-CeO_2_-TiO_2_-Al_2_O_3_-thermally aged (D).

The data from the XRD patterns of the obtained support, fresh, used, after sulfur poisoning and thermally aged catalysts are presented in Figure 4. The diffraction pattern of the support is broad, indicating a relatively low degree of the crystallinity of the phases. In this material, the two phases AlO(OH) (Bohemite)(ICDD PDF 83-2384) together with γ-Al_2_O_3_ (ICDD-PDF- 70-9085) are identified.

The pattern of fresh catalyst demonstrates the presence of the AlO(OH), γ-Al_2_O_3_, and PdO (ICDD-PDF 41-1107). The basic diffraction peak of palladium oxide is very small and broad, suggesting a significant degree of dispersion of the palladium across the catalytic surface. After catalytic tests, the phase composition of the support is changed. AlO(OH) is transformed to γ-Al_2_O_3_, which remains the primary component of the support along with the introduction of a CeO_2_-type phase (ICDD-PDF-81-792).

The PdO phase remains in the used, largely unchanged sample. Nevertheless, a novel, highly crystalline metal Pd phase (ICDD-PDF-46-1043) appears after the catalytic experiments. After the thermal aging at 500 °C for 170 h, the cubic metal palladium phase is not detectable by XRD. The calculated crystallite size of the deposited palladium oxide phase and palladium are provided in Table 2.

Observation reveals that in the fresh and used samples after the sulfur poisoning, the crystalline size of PdO is around 11–12 nm, while after thermal aging, it becomes 19 nm.

### 3.4. Scanning Electron Microscopy

Figure S1 represents the SEM photographs of the studied catalysts. SEM analysis of fresh Pd/La_2_O_3_-CeO_2_-TiO_2_-Al_2_O_3_ reveals an inhomogeneous grainy structure, which after the catalytic test becomes homogeneous. The chemical composition of fresh and used catalytic samples after sulfur poisoning was examined by EDX. The data are presented in Table S1.

The obtained results are in agreement with the applied nominal ratio for the used elements. In the used, after sulfur poisoning of the catalyst, sulfur was detected, which is evidence of the formation of some sulfate or sulfite compounds on the surface of the catalyst after prolonged exposure to SO_2_ and H_2_O.

### 3.5. Transmission Electron Microscopy

**Figure 5:**
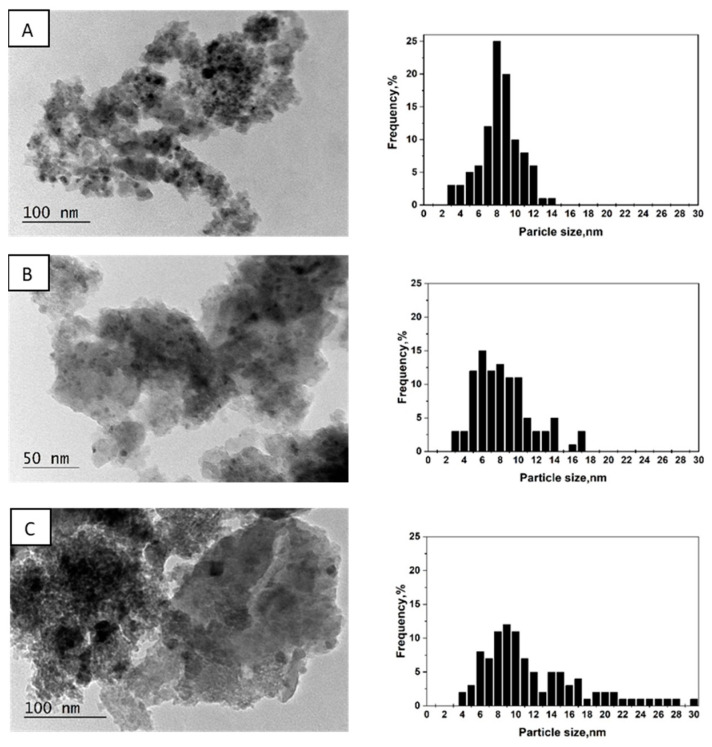
TEM images and corresponding particle size distribution histograms of Pd/La_2_O_3_-CeO_2_-TiO_2_-Al_2_O_3_-fresh (**A**); Pd/La_2_O_3_-CeO_2_-TiO_2_-Al_2_O_3_-used, after sulfur poisoning (**B**) and Pd/La_2_O_3_-CeO_2_-TiO_2_-Al_2_O_3_-thermally aged (**C**).

The morphology and phase composition of Pd/La_2_O_3_-CeO_2_-TiO_2_-Al_2_O_3_ were examined by high resolution transmission electron microscopy (HRTEM). The catalysts exhibit uniform dispersion of palladium, appearing as dark spots across all samples (as depicted in Figure 5). The mean particle size, determined by analyzing 200 randomly chosen nanoparticles in a fresh prepared catalyst, was found to be 8 nm.

After catalytic tests, the average size in Pd/La_2_O_3_-CeO_2_-TiO_2_-Al_2_O_3_ remains the same, which can be evidence of high dispersion, and, therefore, of the thermal stability of Pd particles. After thermal aging, the enlargement of Pd particle size and some agglomerates is observed (Figure 5C). The main crystallite size has increased to 12 nm. Certain research findings [34] have indicated that alteration in the Pd catalyst morphology during the reaction can be impacted by changes in the support materials, potentially leading to an influence on the overall performance of the catalyst.

**Figure 6:**
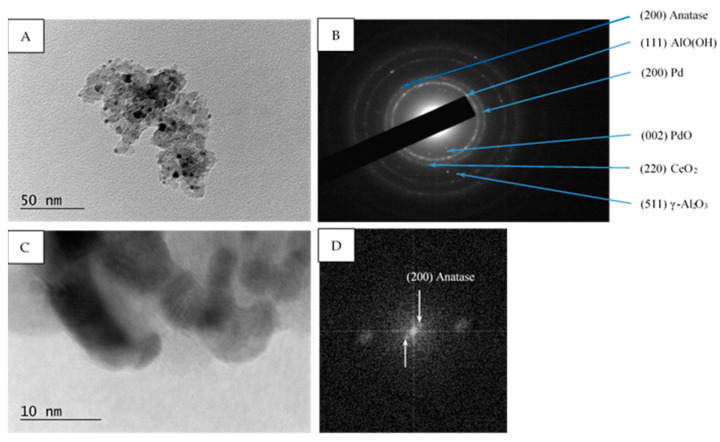
TEM micrograph (**A**), corresponding SAED pattern (**B**), HRTEM micrograph (**C**), and corresponding FFT (Fast Fourier Transform) (**D**) of Pd/La_2_O_3_-CeO_2_-TiO_2_-Al_2_O_3_ fresh catalyst. The identified (200) reflexes correspond to d = 1.8900 Å crystal planes of anatase (PDF 83-2243).

**Figure 7:**
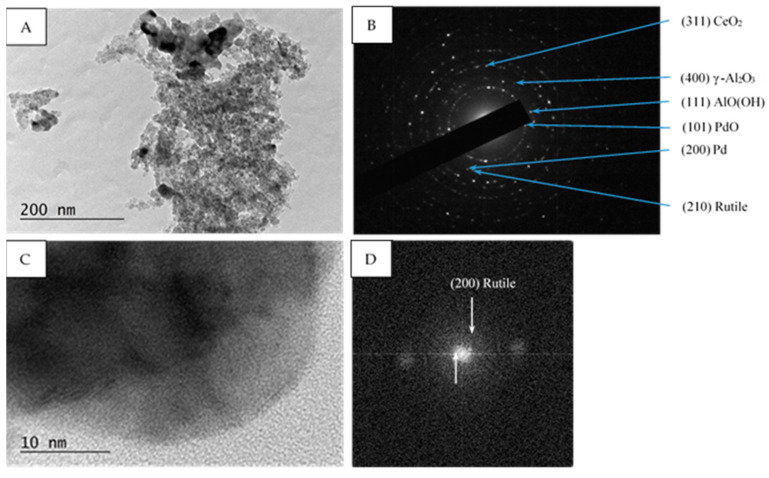
TEM micrograph (**A**), corresponding SAED pattern (**B**), HRTEM micrograph (**C**), and corresponding FFT (**D**) of Pd/La_2_O_3_-CeO_2_-TiO_2_-Al_2_O_3_ used after, sulfur poisoning catalyst. The identified (200) reflexes correspond to d = 2.2968E (Rutile PDF 87-0920).

**Figure 8:**
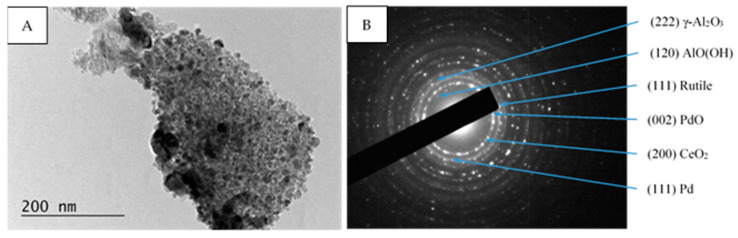
TEM micrograph (**A**) and corresponding SAED pattern (**B**) of sample Pd/La_2_O_3_-CeO_2_-TiO_2_-Al_2_O_3_-thermally aged.

Within all samples (fresh, used, after sulfur poisoning, and thermally aged) the selected area electron diffraction (SAED) patterns show the presence of PdO (PDF-41-1107), Pd (PDF-46-1043), γ-Al_2_O_3_ (PDF-70-9085), AlO(OH) (PDF-83-2384) and CeO_2_ (PDF-81-0792), (Figure 6B, Figure 7B and Figure 8B). Additionally, in the Pd/La_2_O_3_-CeO_2_-TiO_2_-Al_2_O_3_-fresh sample the formation of TiO_2_—anatase (PDF-83-2243) was confirmed by the HRTEM and SAED analyses (Figure 6B,D), while in used, after sulfur poisoning and thermally aged samples, the TiO_2_—rutile (PDF-88-1175) and (PDF-87-0920) was detected (Figure 7B,D and Figure 8B).

### 3.6. X-ray Photoelectron Spectroscopy

**Figure 9:**
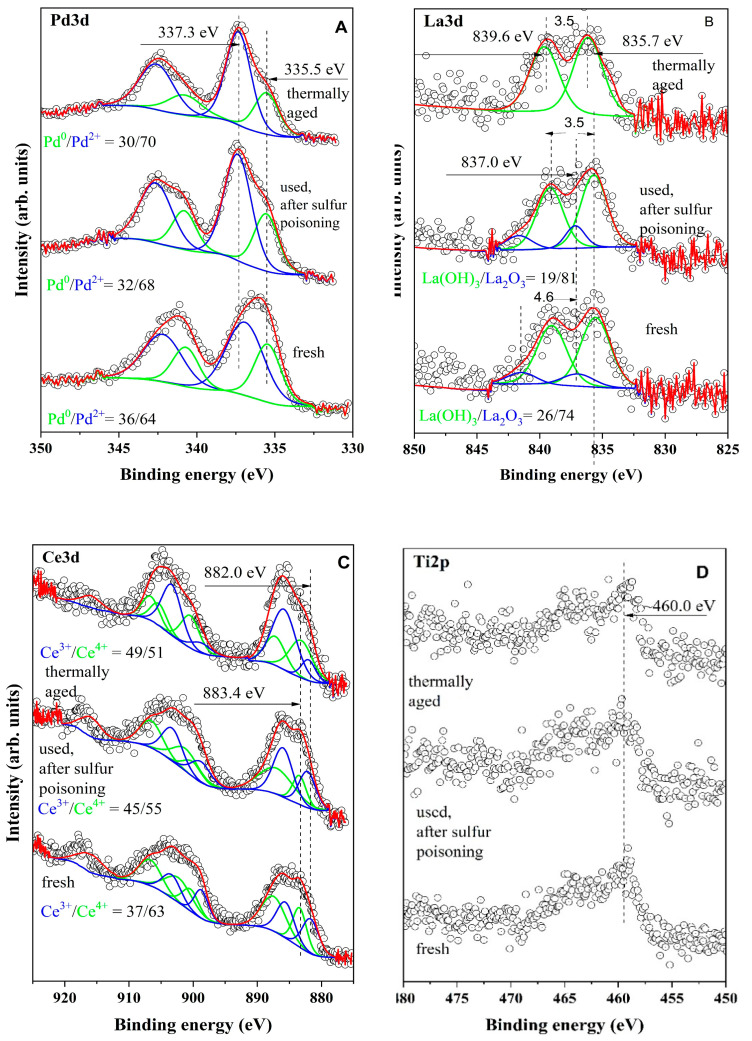
X-ray photoelectron spectra of La3d (**A**), Pd3d (**B**), Ce3d (**C**) and Ti2p (**D**) for Pd/La_2_O_3_-CeO_2_-TiO_2_-Al_2_O_3_-fresh, Pd/La_2_O_3_-CeO_2_-TiO_2_-Al_2_O_3_-used, after sulfur poisoning and Pd/La_2_O_3_-CeO_2_-TiO_2_-Al_2_O_3_-thermally aged. (**A**) The open circles represents experimental data both oxidation states are in green (Pd^0^) and blue (Pd^2+^). The red line is used for resulting curve of curve fitting procedure to be compared with experimental data. (**B**) The open circles represents experimental data. La(OH)_3_ is shown in green, whereas, the blue line is used for La_2_O_3_. The red line is used for resulting curve of curve fitting procedure to be compared with experimental data. (**C**) The open circles represents experimental data both oxidation states are in green (Ce^4+^) and blue (Ce^3+^). The red line is used for resulting curve of curve fitting procedure to be compared with experimental data.

**Figure 10:**
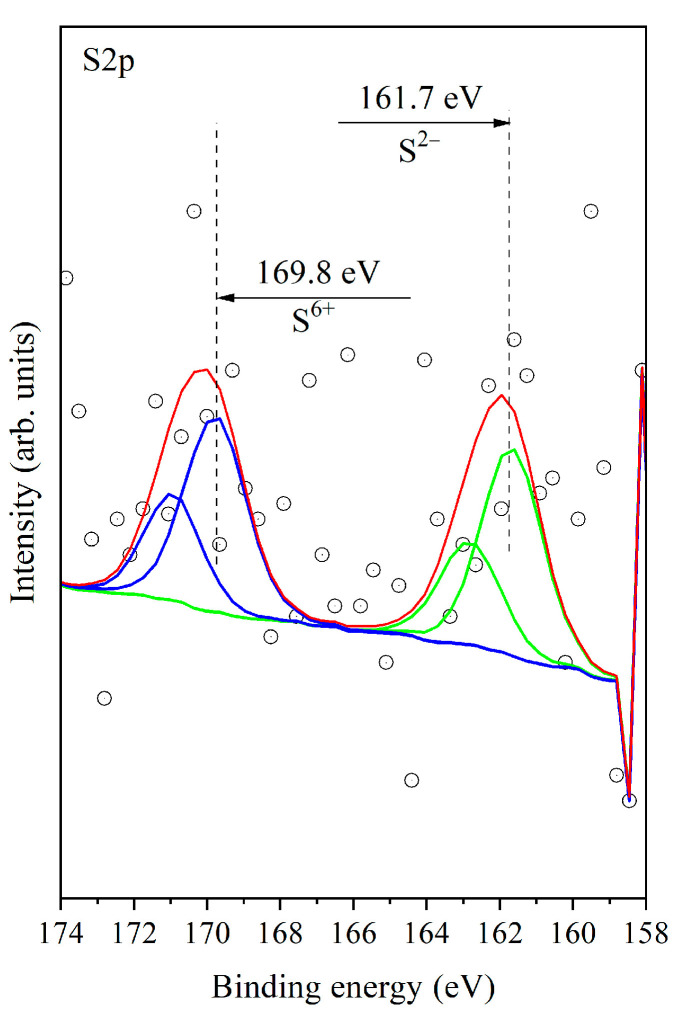
X–ray photoelectron spectra of S2p for Pd/La_2_O_3_-CeO_2_-TiO_2_-Al_2_O_3_-used, after sulfur poisoning catalyst. The open circles represents experimental data both oxidation states are in green (S^2−^) and blue (S^6+^). Because we are measuring 2p core level of sulfur it is doublet peak representing the standard spectra of each oxidation state. The red line is used for resulting curve of curve fitting procedure to be compared with experimental data.

The XPS results are shown in Table 3, Figure 9 and Figure 10. The binding energies for all investigated catalysts are distinguished as follows: in the interval of 335.7–335.1, BE is attributed to Pd^0^ species from metal palladium particles, while in the interval of 337.1–336.3 eV, they are assigned to palladium oxide [35].

As can be seen from Figure 9B, the concentration of Pd^2+^ slightly increases in the used and thermally treated samples. A similar phenomenon was observed in our previous studies [36,37], and this effect is explained by the oxidation of Pd to PdO. In the case of the studied system Pd/La_2_O_3_-CeO_2_-TiO_2_-Al_2_O_3_, the variation of the surface concentration of Pd^2+^ is insignificant, which supports the stability of the catalysis.

According to published data, the 3.5 eV splitting between the primary peak and the satellite of La3d_5/2_ is typical for La(OH)_3_, and the splitting of 4.5 eV is characteristic for La_2_O_3_ (Figure 9A). These results imply that both La_2_O_3_ and La(OH)_3_ are present on the surfaces of both the fresh and used catalysts, after sulfur poisoning catalysts. It is likely that the La_2_O_3_ is covered with La(OH)_3_, as previous research [38] has suggested that lanthanum oxide tends to spontaneously react with water vapor at ambient temperature, resulting in the formation of La(OH)_3_ [39]. In the case of the aged sample, La(OH)_3_ is the only species on the surface.

The XPS spectra in the Ce3d region are presented in Figure 9C. A well-known fact is that some Ce-containing samples are sensitive to X-rays in a vacuum, which leads to a change of the oxidation state of Ce. This complicates quantitative XPS analysis and defining the oxidation state of ceria and eventually the ratio Ce^3+^/Ce^4+^. In our experimental data, we have provided a careful measurement and each scan was recorded separately. After a comparative analysis of the shape of the recorded curves and binding energies, we did not observe any changes between scans. To increase the signal-to-noise ratio, we have performed summation of separate scans. The curve fitting procedure was applied to the obtained spectra to determine their composition and to estimate the quantitative ratio between Ce^3+^ and Ce^4+^ [40]. The curve-fitted XPS spectra of Ce3d are shown in Figure 9C. It can be seen that the concentration of Ce^3+^ increases after aging and after testing with SO_2_. The presence of Ce^3+^ has been proven through EPR spectroscopy (see discussion below). The obtained binding energies for Ti 2p peak for the fresh sample and after the catalytic test in the presence of SO_2_ is at 460 eV. This binding energy is ascribed to Ti^4+^ in TiO_2_ particles.

S2p spectra (Figure 10) show two peaks at ~169.8 eV and ~161.7 eV. The bands with the same positions have been observed in publication [41] when Pt/Al_2_O_3_ reacts with the sulfur dioxide + oxygen mixture and have been attributed to the simultaneous formation of the sulfate species and the sulfide species. In our case, aluminum oxide predominates in the support, and we can attribute the band at ~169.8 eV and ~161.7 eV to the formation of sulfates and the sulfide species as well. The S 2p_3/2_ binding energy typically ranges from 160 eV to 164 eV in metal sulfides [42]. The presence of sulfates is also confirmed by FTIR spectroscopy. Taking the references mentioned above, we can assume that sulfides are formed on the surface of palladium in our case as well.

### 3.7. Electron Paramagnetic Resonance

**Figure 11:**
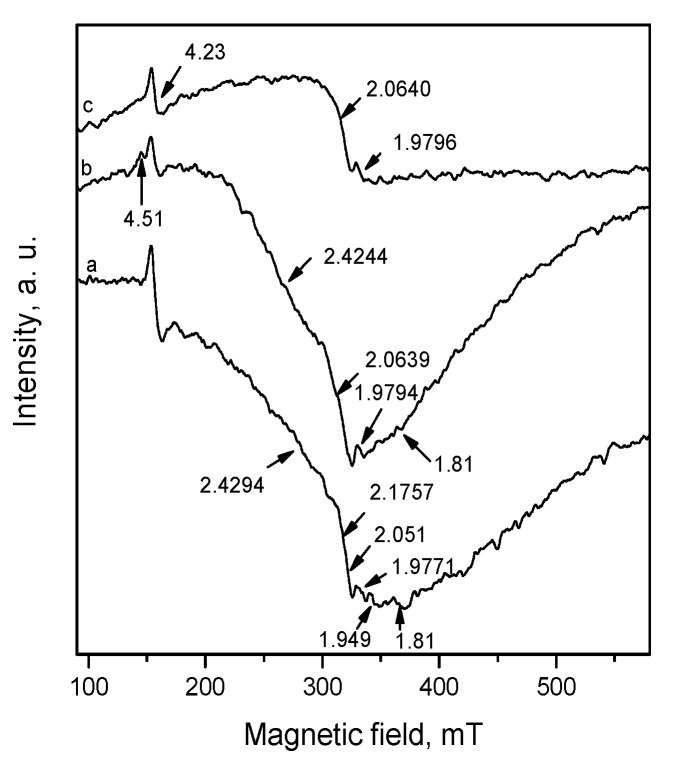
EPR spectra of: (a) Pd/La_2_O_3_-CeO_2_-TiO_2_-Al_2_O_3_-fresh (a); Pd/La_2_O_3_-CeO_2_-TiO_2_-Al_2_O_3_-used, after sulfur poisoning (b) and Pd/La_2_O_3_-CeO_2_-TiO_2_-Al_2_O_3_-thermally aged (c).

The EPR spectra of Pd/La_2_O_3_-CeO_2_-TiO_2_-Al_2_O_3_ catalysts at temperature 123 K are shown in Figure 11.

The EPR spectra are complex and are composed of superposition of several overlapping EPR lines. In all spectra, a line with a g value of 4.23 is recorded. This line is related to Fe^3+^, which very often is contained as an impurity in the starting substances, and it is recorded because of the very high sensitivity of EPR spectroscopy. In the fresh sample (Figure 11a), the EPR lines located at g = 2.4294, 2.1757 and 2.051 are due to the presence of paramagnetic palladium particles, which can be Pd^+^ or Pd^3+^. The g values are slightly different from those reported in the literature probably because of different conditions and the environment of the palladium in the present work [43]. It should be pointed out that the palladium species in the higher than +2 oxidation state were detected by XPS, as reported in our previous investigation [15]. The fact that in this study they were observed only with EPR spectroscopy gives us reason to suggest that they are localized in volume.

The EPR lines with g values 1.9771 and 1.949 are assigned to Ce^3+^ or Ti^3+^. Both ions have similar EPR parameters and the simultaneous presence in the system makes their separation difficult. Two narrow signals with practically the same g values (g_⊥_ = 1.967 and g_II_ = 1.944) are attributed to Ce^3+^ ions associated with an anion vacancy or electrons trapped at anion vacancies partially delocalized onto orbitals of cerium ion [44].

It should be pointed out that similar EPR parameters were reported for Ti^3+^ in anatase [45]. The XPS data show the presence of Ce^3+^. From the XPS data, it can be argued unambiguously that the presence of Ti^3+^ is due to the very low intensity signal in the Ti2p region. Taking into account that the anatase is established by SAED analysis in the fresh sample and EPR is a highly sensitive technique that allows investigation of paramagnetic species [46]; thus, we cannot exclude the presence of Ti^3+^.

The EPR lines due to Ce^3+^ or Ti^3+^ in the fresh sample, in the EPR spectra after thermal aging are maintained and positioned at g factor 1.9796 (Figure 11c). This shows that the paramagnetic ions, which are responsible for it are located in the volume. In addition, a weak signal with g = 1.81 is detected, which, according to the literature data, is connected with Ce^3+^ in close range to La [47]. Ce^3+^, as a 4f^1^ ion, is characterized by strong spin-orbital coupling leading to large deviations from the g factor of free electron (2.0023). Moreover, due to the short relaxation times, it is detectable usually at low temperatures. The EPR line with g = 1.81 disappears after thermal aging, which proves that these Ce ions are on the surface of La_2_O_3_.

After catalytic tests, the EPR spectra do not change significantly. Again, signals for palladium paramagnetic ions are observed, but this time the temperature dependence of the signal with g 2.4244 shows the presence of the superparamagnetic palladium particles.

**Figure 12:**
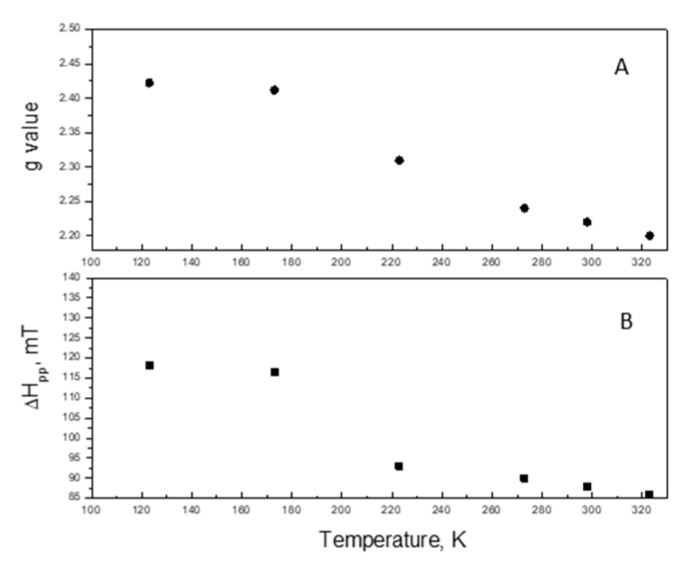
The variation of the g factor (**A**) and line width (**B**) of the EPR spectra changing in the recording temperature.

This is because, with the decrease of the recording temperature, the EPR line is broadening and is moved to the lower magnetic field (Figure 12). This behavior is typical for superparamagnetic particles. Nonlinear behavior shows that the particles have various sizes but the superparamagnetic state remains. In addition, a line with a g value of 4.51 is recorded, which is due to Ce^3+^. This fact, together with the increase in the intensity of the EPR signal with g = 1.9794, shows that during the reaction, a reduction of the cerium ions is taking place.

### 3.8. FTIR Spectroscopy

**Figure 13:**
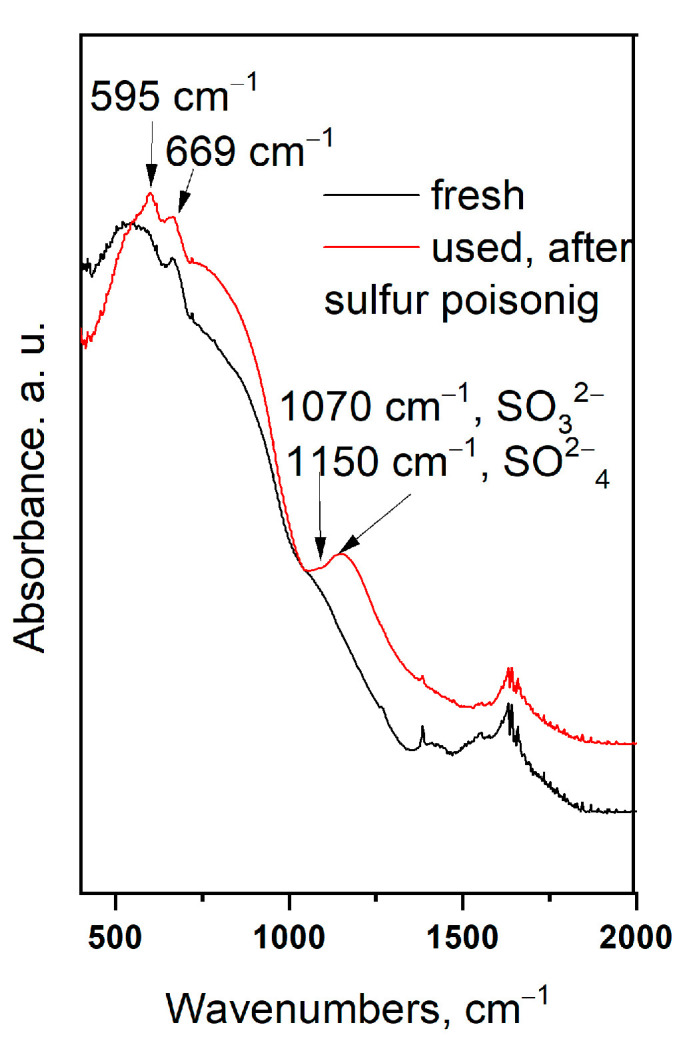
Infrared spectra of Pd/La_2_O_3_-CeO_2_-TiO_2_-Al_2_O_3_-fresh and Pd/La_2_O_3_-CeO_2_-TiO_2_-Al_2_O_3_-used, after sulfur poisoning catalysts.

FTIR spectra of a fresh Pd/La_2_O_3_-CeO_2_-TiO_2_-Al_2_O_3_ sample and after the catalytic test with sulfur dioxide are presented in Figure 13. Low-resolved bands centred at 1150 cm^−1^ and 1070 cm^−1^ are visible in the IR spectra. The band centred at 1150 cm^−1^ is ascribed to the sulphates in bulk according to data in the literature [48]. There is no evidence of the presence of aluminium sulfate, either X-ray or TEM, which can be considered as evidence that the formed sulphates are amorphous.

The band at 1070 cm^−1^ is very weak and strongly overlaps with the band of the support. According to Schoonheydt [49], the vibration at 1070 cm^−l^ is assigned to a SO_3_^2−^ species coordinated through its sulfur.

In this investigation, as in the previous publication [15], no band was observed at 1435 cm^−1^ for sulphate groups formed on the palladium particles. The adsorption bands at 595 cm^−1^ and 669 cm^−1^ most likely are the result of PdO [50,51].

### 3.9. Reaction Kinetics

To extend further the analysis of the studied catalysts, an investigation on the kinetics and mechanism of the reaction has been carried out. The kinetics parameters calculations were performed by multivariate analysis. For these calculations, data from the temperature-conversion curves were used for direct integration of the reaction rates. To fit the experimentally measured rates with kinetics parameters, a special computation program for a numerical (nonlinear) optimization was used. The minimized residual sum of squares between the measured experimental points and the corresponding predictions of the model (RSS) and the squared correlation coefficient (R^2^) were selected as optimization criteria for the model’s consistency. Details on the calculation procedure were published earlier [52,53].

The values for the calculated reaction order towards the oxygen lead to the suggestion of a significant role of the oxygen chemisorption (Table 4, Power law kinetics model). The reaction order towards the water vapor (−0.33) reveals a very significant inhibition effect.

**Figure 14:**
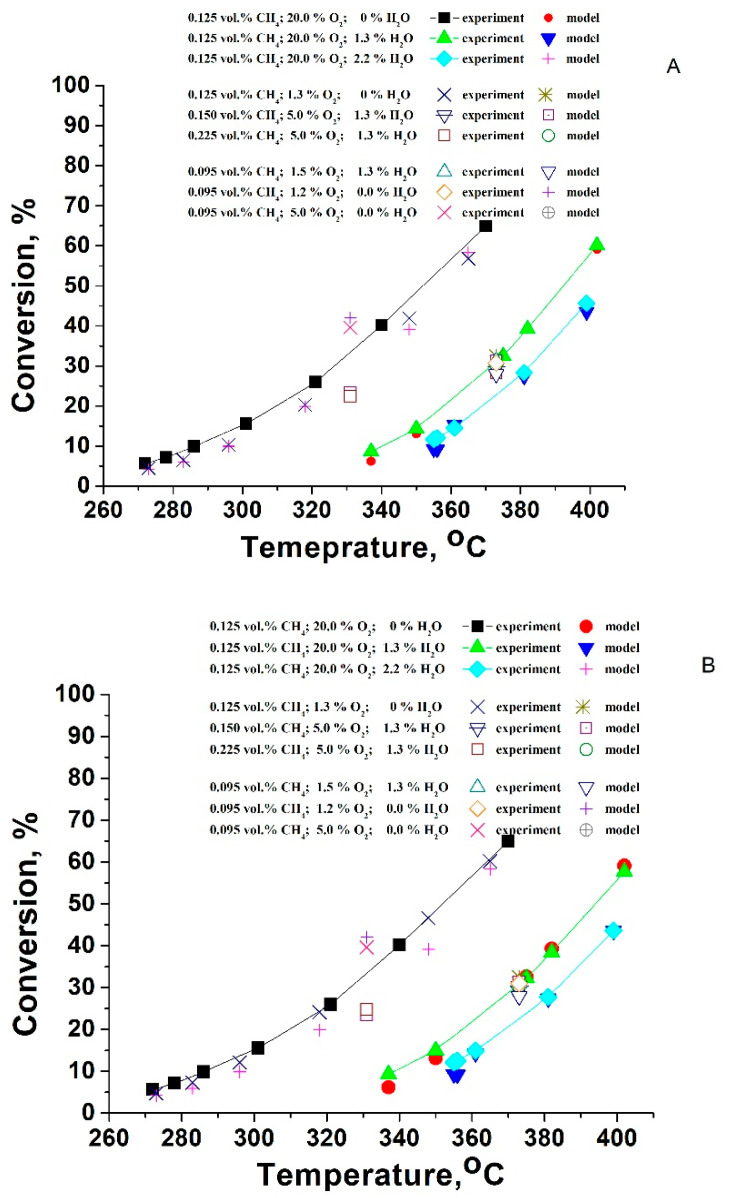
Comparison between the experimentally measured conversions at different conditions and the model prediction by the Mars–van Krevelen mechanism (**A**) and Langmuir–Hinshelwood mechanism (**B**).

The relevance of the mechanistic models used for the kinetic calculations towards the experimental data set is presented in Figure 14 and the calculated results are given in Table 5 and Table 6. One could see that the lowest values for RSS criteria and the highest correlation between the model and experiment are obtained for the Mars–van Kerevelen model with the assumption that the water adsorbs on oxidized and reduced sites and slow desorption of products occur (MVK-SDP). Therefore, this mechanism should be considered as more consistent with the experimental results than the alternative Langmuir–Hinshelwood mechanism, where the water competes with oxygen and methane.

Summarizing, the deposition of Pd to the La_2_O_3_- CeO_2_- TiO_2_-Al_2_O_3_ system leads to a decrease in the specific surface area, accompanied by an increase in the average pore diameter from 4.9 nm to about 8 nm, the pore-size distribution being transformed from a mono- to bimodal structure. Based on the literature data [37], this morphological structure offers significant benefits when these materials are used as catalytic support. More specifically, the small pores ensure a large surface area for better dispersion of the supported active phase (noble metal, for instance). The larger pores provide conditions for improved internal mass transfer within the catalyst.

As reported [54], catalyst thermal deactivation can occur due to several factors: (i) reduction in the active surface area due to the enlargement of palladium particles, (ii) pore collapse of the active phase; (iii) decrease in the catalytic support area; and (iv) alterations in the chemical composition of active catalytic phases into less active phases.

Typically, the sintering occurs at high temperatures (>500 °C) and is accelerated in the presence of water vapor [55], the driving force being the minimization of the surface energy, reduced by the transport and increase in the particles [56]. Within our study, the thermal deactivation behavior of the catalyst may be explained by phase changes of TiO_2_. It is known that anatase irreversibly transforms to rutile at elevated temperatures. This transformation does not have a fixed temperature. Pure bulk anatase is transformed irreversibly to rutile in air at 600 °C; however, the reported transition temperatures vary in the range 400–1200 °C [57,58,59], owing to the use of different methods of determining the transition temperatures, raw materials, and processing methods.

During the heat treatment part of TiO_2_, anatase may be transformed to rutile and the rutile grains coarsen at the expense of neighbouring anatase until the large rutile grains begin to impinge on each other [60,61]. This increase in grain size leads to a decrease in surface area and a further decrease in activity [62,63,64]. Additionally, it is reported that calcination above 465 °C has always resulted in the phase rutile [65]. The phase transition is associated with increased crystal size, resulting in a significant decrease in specific surface area [66].

In our case, the concentration of TiO_2_ (8 wt.%) is not sufficient for reliable XRD analysis, and the obtained XPS data show low intensity broad peaks, the only possible determination of the changes was made by HRTEM. Within the present study, the results from HRTEM analysis reveal that the decrease in the specific surface is connected with the transformation of part of the anatase to larger particles of rutile (whose process is reported to proceed at temperatures above 465 °C [65].

In parallel, the deactivation could be related to the growth of the palladium particles from 8 nm to 12 nm and the increased PdO/Pd ratio, more specifically, this ratio is higher than its optimal value for the applied reaction conditions. As reported by Su at all [67], small palladium particles enhance the activity of PdO in methane combustion by facilitating the dissociation of CH_4_ without being oxidized under the given reaction conditions. It should be pointed out that the presence of metallic Pd in contact with PdO facilitates the reduction of PdO by CH_4_, i.e., methane activation proceeds more on metallic palladium than palladium oxide [15]. Pd plays a crucial role in dissociating CH_4_ more effectively when compared with PdO, with the resulting reaction products diffusing towards the Pd—PdO interface, where PdO is converted into metallic Pd.

At the same time, the modification with TiO_2_ of the La_2_O_3_-CeO_2_-Al_2_O_3_ system results in higher resistance towards the presence of SO_2_ due to prevailed formation of unstable surface sulfites instead of the thermally stable sulfates, as observed with the system without TiO_2_.

### 3.10. Monolithic Reactor Tests and Modeling

The methane combustion processes were described using a two-dimensional heterogeneous model of a monolithic channel. The conversion degree at the outlet of the monolithic channel was calculated by using the method of mixing-cup average concentration. It consists of multiplying the concentrations of the laminar streamlines by the corresponding volumetric flows and summing up over all the streamlines and dividing this sum by the total volumetric flow. The reported results are for calculated conversion degree and temperature profiles inside the monolithic channel using the obtained data for reactions in cases of isothermal (experiment) conditions and then the behavior of the reactor at adiabatic reactor operation is based on simulation by using the reactor model. The heat transfer within the entire monolithic element ocurrs by conduction trough the channels in the radial direction and by fluid convection in the axial direction. The catalytic element is modeled by assuming that the heat is transferred trough a thin thermal boundary layer with a driving force, proportional to the difference between the temperature in the first to the wall channel and the temperature of the reactor wall. Of course, in the theoretical case, one could suppose complete thermal insulation and the behavior of the monolithic reactor is fully adiabatic. However, in most of the applications, one should expect some extent of heat exchange with the ambient environment and therefore, the effect of the wall temperature has been taken into account. For convenience, the temperature and concentration profiles are colored according to the calculated values, i.e., from blue color for low temperatures or conversions towards the red color for their high values (passing through mixed colors within the intermedia values). A second-order approximation is used for the numerical solution; further details are provided in the literature [68,69,70,71,72,73].

**Figure 15:**
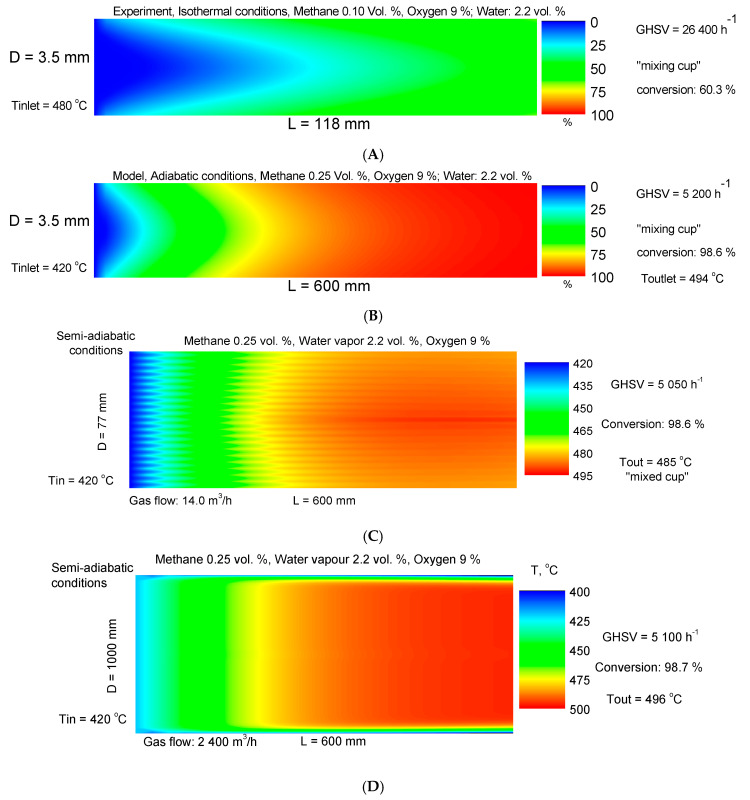
Experimentally measured conversion degrees and temperature profiles in a single monolithic channel at isothermal conditions (**A**); simulated conversion degrees and temperature profiles within a single adiabatic channel for ensuring of 99% methane conversion (**B**); pilot-scale simulation of methane combustion using the kinetic data, obtained at isothermal conditions (**C**); and full-scale reactor model for methane combustion accouning for the heat loss at the reactor wall (**D**).

The simulation results (Figure 15) demonstrate the possibilities for the abatement of 2400 Nm^3^/h methane-containing gas (CH_4_: 0.25 vol.%, H_2_O: 2.2 vol.%, 9 vol.% O_2_). The model prediction shows that the required dimensions of the monolith for achieving 99% conversion are the following: D = 1.0 m and L = 0.6 m. Therefore, for effective neutralization of methane in presence of water, the reactor should operate adiabatically at GHSV of 5100 h^−1^.

The practical significance of these results is closely tied to challenges arising from mobile sources of pollution, specifically stemming from the release of unburned hydrocarbons in the exhaust emissions of internal combustion engines utilizing natural gas (primarily composed of approximately 95% methane) as their fuel source. Consequently, there exists a notable interest in the development of novel catalytic converter materials that can ensure highly efficient reduction of methane emissions.

## 4. Conclusions

The deposition of palladium to the La_2_O_3_-CeO_2_-TiO_2_-Al_2_O_3_ system leads to a decrease in the specific surface area, accompanied by an increase in the average pore diameter, with the pore-size distribution transforming from a mono- to bimodal structure. The methane complete oxidation reaction occurs at temperatures exceeding 220 °C. T_50_ in the absence of water vapor is 328 °C. However, in the presence of 1.2% water vapor (with 5% oxygen content and GHSV_STP_ of 60,000 h^−1^), T_50_ increases to 370 °C.

Most likely, the reaction of complete oxidation of methane follows the Mars−van Krevelen mechanism, where the water molecules adsorb on both oxidized and reduced sites. A slow desorption of the products (CO_2_, H_2_O) is also suggested and implemented into the rate equation.

The thermal aging at 500 °C leads to lowering the catalytic activity, which is due to the (i) agglomeration of the Pd-clusters (from 8 nm to 12 nm); (ii) transformation of part of the TiO_2_ from anatase to larger particles of rutile, resulting in a decreased specific surface area; and (iii) increased ratio PdO/Pd above its optimal value, which is specific for the applied reaction conditions. The presence of Ce^3+^ on the catalytic surface and in the volume was revealed by EPR and XPS. Most likely, the Ce^3+^ ions on the surface are near lantana.

The modification with Ti leads to improved activity in the presence of water vapor after thermal aging and a slightly decrease in the dry gas mixture. The effect of water vapor in terms of calculated reaction order is −0.33.

The simultaneous formation of sulfats, sulfites, and sulfides in the studied system is suggested. The benefit of the modification with TiO_2_ of the La_2_O_3_-CeO_2_-Al_2_O_3_ system is the higher resistance towards the presence of SO_2,_ most likely due to the prevailing formation of unstable surface sulfites instead of the thermally stable sulfates, as observed with the system without TiO_2_.

The results from the kinetic model calculation show that the reaction pathway over the Pd/La_2_O_3_-CeO_2_-TiO_2_-Al_2_O_3_ catalyst follows the Mars–van Krevelen mechanism. A sample of Pd/La_2_O_3_-CeO_2_-TiO_2_-Al_2_O_3_, supported on rolled stainless steel with aluminum content (Aluchrom VDM^®^), was produced and tested to assess the potential practical applications of the obtained material. A two-dimensional heterogeneous model of a monolithic channel was employed to simulate methane oxidation within an industrial scale monolithic reactor.

## Figures and Tables

**Table 1:** Specific surface areas and pore properties of La_2_O_3_-CeO_2_-TiO_2_-Al_2_O_3_ support and Pd/La_2_O_3_-CeO_2_-TiO_2_-Al_2_O_3_ catalysts.

Sample	S_BET_ m^2^/g	S_ext_ m^2^/g	S_micro_ m^2^/g	V_micro_ cm^3^/g	Total Pore Volume cm^3^/g	Average Pore Diameter nm
La_2_O_3_-CeO_2_-TiO_2_-Al_2_O_3_-support	210	134	76	0.04	0.26	4.9
Pd/La_2_O_3_-CeO_2_-TiO_2_-Al_2_O_3_-fresh	106	103	3	0.02	0.19	7.4
Pd/La_2_O_3_-CeO_2_-TiO_2_-Al_2_O_3_-used, after sulfur poisoning	100	-	-	-	0.22	8.6
Pd/La_2_O_3_-CeO_2_-TiO_2_-Al_2_O_3_-thermally aged	82	-	-	-	0.16	8.1

**Table 2:** Phase composition and average crystallite size.

Sample	Phase Composition According to XRD	Phase Composition According to HRTEM	PdO (nm) ^a^	Pd (nm) ^a^	Mean Crystalline Size (nm) ^b^
La_2_O_3_-CeO_2_-TiO_2_-Al_2_O_3_-support	AlO(OH), γ-Al_2_O_3_,	-	-	-	
Pd/La_2_O_3_-CeO_2_-TiO_2_-Al_2_O_3_-fresh	AlO(OH), γ-Al_2_O_3_, PdO	AlO(OH), γ-Al_2_O_3_, PdO, Pd, CeO_2_, TiO_2_-anatase	12	-	8
Pd/La_2_O_3_-CeO_2_-TiO_2_-Al_2_O_3_-used, after sulfur poisoning	γ-Al_2_O_3_, CeO_2_,PdO, Pd	AlO(OH), γ-Al_2_O_3_, PdO, Pd, CeO_2_, TiO_2_-rutile	11	40	8
Pd/La_2_O_3_-CeO_2_-TiO_2_-Al_2_O_3_-thermally aged	AlO(OH), γ-Al_2_O_3_, PdO	AlO(OH), γ-Al_2_O_3_, PdO, Pd, CeO_2_, TiO_2_-rutile	19	-	12

^a^ Crystallite size calculated by the size-strain analysis tool implemented in the Topas 4.2 program. ^b^ Determinate from TEM.

**Table 3:** Surface atomic concentration, at.%.

Sample	O1s	Al2p	La3d	Ce3d	Pd3d	Ti2p	S2p
S2Pd/La_2_O_3_-CeO_2_-TiO_2_-Al_2_O_3_-fresh	56.48%	36.14%	0.53%	1.45%	4.15%	1.24%	-
Pd/La_2_O_3_-CeO_2_-TiO_2_-Al_2_O_3_-used, after sulfur poisoning	58.40%	31.63%	0.53%	1.38%	6.43%	1.33%	0.30%
Pd/La_2_O_3_-CeO_2_-TiO_2_-Al_2_O_3_-thermally aged	60.00%	30.02%	0.49%	1.41%	6.68%	1.41%	-

**Table 4:** Kinetics parameters based on power law model.

**PWL** $r=kC_{voc}^{m}C_{ox}^{n}C_{water}^{p}$							
	**E_a_**	**k_o_**	**m (CH_4_)**	***n* (O_2_)**	***p* (H_2_O)**	**RSS**	***R* ^2^**
Pd/La_2_O_3_-CeO_2_-TiO_2_-Al_2_O_3_	108.0	3.65 × 10^9^	0.94	0.02	−0.33	10.6	1.00

E_ai_, kJ/mol; k_oi_, mol.s^−1^.m^−3^; k_oi,pwl_, mol.s^−1−[1−(m+*n*+p)]^; E_ai_, kJ/mol.

**Table 5:** Reaction rate expressions and kinetics parameters for applied MVK-SDP model.

**Model: MVK-SDP, (Water Adsorbs on Oxidized and Reduced Sites, Slow Desorption of Products)** $r=\frac{k_{red}k_{ox}C_{voc}C_{ox}^{}}{\gamma k_{red}C_{voc}\left(1+K_{water-voc}.C_{water-voc}\right)+k_{ox}C_{ox}^{}\left(1+K_{water-ox}.C_{water-ox}\right)+(k_{red}k_{ox}/k_{des})C_{voc}C_{ox}^{}}$γ = 2												
	**E_a_._ox_**	**k_o_._ox_**	**E_a_._red_**	**k_o_._red_**	**−ΔH_w_._ox_**	**k_o_._water_. _ox_**	**ΔH_w_._red_**	**k_o_._w, red_**	**E_a_._des_**	**k_o_._des_**	**RSS**	**R^2^**
Pd/La_2_O_3_-CeO_2_-TiO_2_-Al_2_O_3_	124.1	4.49 × 10^11^	51.3	1.02 × 10^6^	94.8	3.01 × 10^−7^	74.6	1.89 × 10^−7^	95.8	1.56 × 10^8^	3.8	1.00

E_ai_, kJ/mol; ΔH_i_. kJ/mol; k_oi_, m^3^/mol; k = k_o_. exp(−E_a_/RT); K_i(voc,ox, w)_ = k_o(voc,ox, w)_. exp(−ΔH_i_,_voc,ox, w_/RT); −ΔH_i_ =E_des_ − E_ads_.

**Table 6:** Reaction rate expressions and kinetics parameters for applied Langmuir−Hinshelwood model.

**LH-DS-D: water compete with oxygen and methane** $r=\frac{kK_{voc}C_{voc}K_{ox}^{1/2}C_{ox}^{1/2}}{\left(1+K_{voc}C_{voc}+K_{water-voc}C_{water})(1+K_{ox}^{1/2}C_{ox}^{1/2}+K_{water-ox}C_{water}\right)}\;$												
	***E_a_***	***k_o_***	**−Δ*H_voc_***	***k_o_._voc_***	**−Δ*H_ox_***	***k_o_._ox_***	**Δ*H_water_***	***k_o_._water_***	**Δ*H_water_._red_***	***k_o_._water_. _red_***	**RSS**	**R^2^**
Pd/La_2_O_3_-CeO_2_-TiO_2_-Al_2_O_3_	136.6	6.60 × 10^11^	169.2	1.22 × 10^3^	77.4	5.23 × 10^−6^	79.5	7.96 × 10^−1^	75.8	8.08 × 10^−6^	4.8	0.98

E_ai_, kJ/mol; ΔH_i_. kJ/mol; k_oi_, m^3^/mol; k = k_o_. exp(−E_a_/RT); K_i(voc,ox, w)_ = k_o(voc,ox, w)_. exp(−ΔH_i_,_voc,ox, w_/RT); −ΔH_i_ = E_des_ − E_ads,_ RSS—squared sum of residuals. R^2^—squared correlation coefficient.

## Data Availability

Data sharing is not applicable to this article.

## Supplementary Materials

The following supporting information can be downloaded at: https://www.mdpi.com/article/10.3390/ma16206784/s1, Figure S1. SEM images of (a) Pd/La_2_O_3_-CeO_2_-TiO_2_-Al_2_O_3_-fresh and (b) Pd/La_2_O_3_-CeO_2_-TiO_2_-Al_2_O_3_-used, after sulfur poisoning catalysts and Table S1. Surface composition from the area of Pd/La_2_O_3_-CeO_2_-TiO_2_-Al_2_O_3_ catalysts obtained by SEM/EDX analysis.
