# Supplementary material for: Effect of TiO2 on Pd/La2O3-CeO2-Al2O3 Systems during Catalytic Oxidation of Methane in the Presence of H2O and SO2
Source: Materials (Basel). 2023 Oct 20;16(20):6784. doi: 10.3390/ma16206784 (PMC10608221; doi:10.3390/ma16206784)
Supplement: Supplementary file 1 [file materials-16-06784-s001.zip › materials-2647075-supplementary.pdf]

Effect of TiO_2 on Pd/La_2O_3-CeO_2-Al_2O_3 Systems during Catalytic Oxidation of Methane in the Presence of H_2O and SO_2

Ralitsa Velinova [1], Silviya Todorova [2], Daniela Kovacheva [1], Hristo Kolev [2], Yordanka Karakirova [2], Pavel Markov [1], Katerina Tumbalova [1], Georgi Ivanov [1] and Anton Naydenov [1,*]

[1] Institute of General and Inorganic Chemistry, Bulgarian Academy of Sciences, Acad. G. Bonchev Str., bl. 11, 1113 Sofia, Bulgaria
[2] Institute of Catalysis, Bulgarian Academy of Sciences, Acad. G. Bonchev Str., bl. 11, 1113 Sofia, Bulgaria
* Correspondence: naydenov@svr.igic.bas.bg

Supplementary Data

1. *SEM/EDX analysis of fresh and used, after sulfur poisoning Pd/La_2O_3-CeO_2-TiO_2-Al_2O_3 catalyst.*

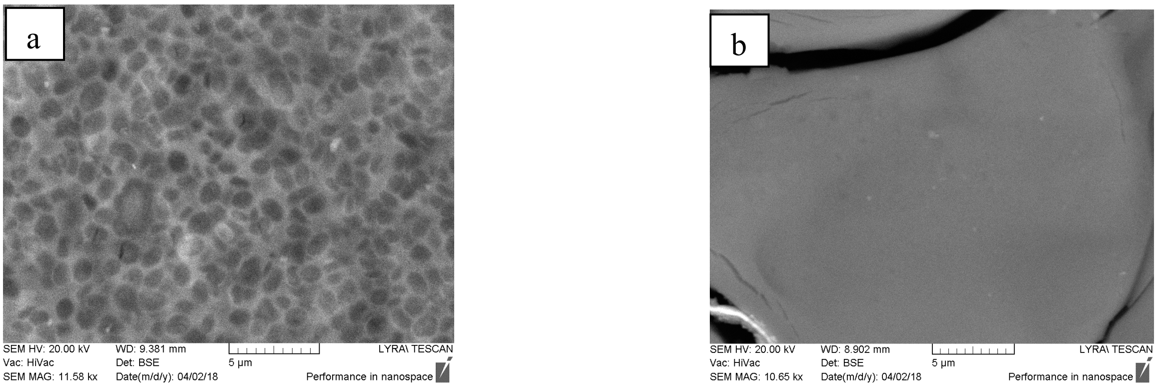

Figure S1. SEM images of a) Pd/La_2O_3-CeO_2-TiO_2-Al_2O_3-fresh and b) Pd/La_2O_3-CeO_2-TiO_2-Al_2O_3 used after sulfur poisoning catalysts.

Table S1. Surface composition from area of Pd/La_2O_3-CeO_2-TiO_2-Al_2O_3 catalysts obtained by SEM/EDX analysis.

Catalyst	O (wt %)	Al (wt %)	Ce (wt %)	La (wt %)	Ti (wt %)	Pd (wt %)	S (wt %)
Pd/La_2O_3-CeO_2-TiO_2-Al_2O_3-fresh	41.3	41.6	8.2	4.5	1.5	2.8	
Pd/La_2O_3-CeO_2-TiO_2-Al_2O_3 used after sulfur poisoning	45.6	35.3	6.8	3.9	0.5	5.9	1.9
